# Lifetime Dependent Variation of Stress Hormone Metabolites in Feces of Two Laboratory Mouse Strains

**DOI:** 10.1371/journal.pone.0136112

**Published:** 2015-08-18

**Authors:** Thomas Kolbe, Rupert Palme, Alexander Tichy, Thomas Rülicke

**Affiliations:** 1 Biomodels Austria, University of Veterinary Medicine Vienna, Vienna, Austria; 2 IFA-Tulln, University of Natural Resources and Life Sciences, Tulln, Austria; 3 Unit of Physiology, Pathophysiology and Experimental Endocrinology, University of Veterinary Medicine Vienna, Vienna, Austria; 4 Bioinformatics and Biostatistics Platform, University of Veterinary Medicine Vienna, Vienna, Austria; 5 Institute of Laboratory Animal Science, University of Veterinary Medicine Vienna, Vienna, Austria; University of Lübeck, GERMANY

## Abstract

Non-invasive measurement of stress hormone metabolites in feces has become routine practice for the evaluation of distress and pain in animal experiments. Since metabolism and excretion of glucocorticoids may be variable, awareness and adequate consideration of influencing factors are essential for accurate monitoring of adrenocortical activity. Reference values are usually provided by baselines compiled prior to the experiment and by age matched controls. The comparison of stress hormone levels between animals of different ages or between studies looking at hormone levels at the beginning and at the end of a long term study might be biased by age-related effects. In this study we analyzed fecal corticosterone metabolites (FCM) during the lifetime of untreated female mice of the strains C57BL/6NCrl and Crl:CD1. For this purpose feces for each individual mouse were collected every two months over a period of 24 hours, at intervals of four hours, until the age of 26 months. Results of the study revealed that age of the animals had a significant impact on the level and circadian rhythm of stress hormone metabolites. Furthermore, long-term observation of mice revealed a strain specific excretion profile of FCM influenced by strong seasonal variability.

## Introduction

Since the non-invasive measurement of hormone metabolites in fecal samples was approved as an alternative for the analysis of plasma glucocorticoid concentrations, it has become a widely accepted technique to diagnose stress response in many animal species (reviewed in [1, 2]). In laboratory rodents for example this approach avoids the stressful blood sampling procedure, which may interfere with an animal's endocrine status. The acute stress response, i.e. the significant increase of glucocorticoid concentration in the blood, often induced by the necessary fixation of an animal and the puncture of a blood vessel, may mask the real physiological condition. In contrast, the analysis of fecal corticosterone metabolites (FCM) to monitor adrenocortical activity allows for frequent sampling without any disturbance to the animals [3]. However, before such a refined experimental method can be reliably applied, detailed knowledge about possible factors, which may influence glucocorticoid metabolite excretion in feces, is essential.

Corticosterone is the major glucocorticoid in mice and therefore it is widely used as an indicator of pain and distress [4]. Supportive for the monitoring of stress hormones via metabolites is that in mice corticosterone metabolites are predominantly excreted via feces [3]. Collection of feces eliminates any methodological problem related to the strong and immediate endocrine response of mice to blood sampling, which is an important external stressor. However, there are also several other factors, which can potentially act on the corresponding endocrine variables, such as the excretion of corticosterone metabolites under unstressed or stressful conditions.

As common for most hormones the secretion of corticosterone and excretion of its metabolites also follow nycthemeral cycles [3, 5]. Moreover, the excretion pattern of corticosterone metabolites strongly depends on the time of day, resulting in a delay of excretion during the light phase, which is characterized as the time of low activity in nocturnal animals [6]. Therefore, a permanent light-dark rhythm and a specified time for sampling should be selected in order to measure reproducible and comparable experimental data [6, 7].

In addition to circadian rhythmicity differences between female and male mice are also reported to be a concern in the fecal excretion of glucocorticoid metabolites. In contrast to females, male mice excrete proportionally more corticosterone metabolites via their feces, however, overall concentration of fecal corticosterone metabolites is significantly higher in females than in males [3, 6]. Sex differences in stress response, measured as plasma corticosterone levels, are significantly influenced by genetic factors, which result in strain specific differences. These differences are readily seen in standard and recombinant inbred strains [8, 9]. A significant increase in plasma corticosterone concentration was found in unstressed late pregnant mice [10], suggesting an estrogen enhanced adrenocortical sensitivity to ACTH during late pregnancy, similarly to rats where this condition has also been observed [11].

In experiments where food is not standardized or constant, the influence of diet composition and preparation should be considered when corticosterone metabolites are measured as concentration in feces. Feeding of high energy diets will reduce fecal mass excreted by the animals, which may bias readings and result in overestimation of stress hormone secretion [12, 13]. In contrast, permanent food restriction or feeding of a calorie restricted diet may induce physiological stress in rodents, resulting in a daily period of mild hyperadrenocorticism, which can be measured as elevated levels of plasma corticosterone and FCM concentrations [14, 15].

Recent findings about bidirectional communication between the brain and the gut demonstrated accumulating evidence for the significance of postnatal microbial colonization as an environmental determinant for the development of anxiety behavior [16]. Axenic (germfree) mice display lower levels of anxiety and increased motor activity, compared to SPF (specific pathogen free) animals with a commensal microflora. The pattern of both behaviors were ‘normalized’ by perinatal exposure of germfree newborns to microbiota obtained from SPF mice [17]. Moreover, ingestion of probiotics, such as lactic acid bacteria, appeared to be beneficial to host physiology, which included a reduction of stress-induced corticosterone levels in normally colonized, healthy mice [18]. In contrast, other research has shown that exposure of neonatal rats to Gram-negative bacterial endotoxin results in chronically elevated basal levels, as well as, a higher and prolonged increase of stress induced corticosterone levels in adulthood [19]. These results suggest that exposure to pathogens in early life has a long-term negative impact on neuroendocrine regulation of stress in adult rodents. Such response may be readily seen in rodents born and raised under inadequate hygienic conditions and supports the need for SPF status of laboratory rodents as standard practice to generate reproducible and comparable experimental results.

Changes of basal corticosterone secretion in response to seasonal changes are considered unlikely in laboratory rodents since these animals are under strictly controlled environmental conditions. Furthermore, domestication in a highly standardized vivarium seems to additionally reduce seasonality in laboratory mice compared to wild-caught mice as shown for parameters of reproduction [20]. Indeed, comparable values of basal serum corticosterone were reported in unstressed C57BL/6J mice in spring and autumn [21]. However, the corticosterone secretion of stressed animals differed significantly between the seasons, suggesting a circannual rhythmicity of the adrenal gland reactivity to stressors [21, 22]. Moreover, observed seasonal changes in pain-related behaviors may result from diurnal variations in the activity of nociceptive systems in laboratory mice [20]. Although the underlying chronobiological mechanisms are not yet identified, melatonin (and its ability to entrain neuro-endocrine rhythms) is suggested as a candidate for triggering circannual changes in pain response of laboratory rodents [23, 24].

Compared to circadian variations annual periodicity of corticosterone secretion in laboratory mice is still poorly investigated. Moreover, there is a lack of information on how basal levels of corticosterone or its metabolites in feces of mice fluctuate over their lifetime. Here we present the results of a long term study of two commonly used laboratory mouse strains to determine the concentrations of fecal corticosterone metabolites over their complete life span.

## Materials and Methods

### Animals

Female mice of the strains C57BL/6NCrl (B6) and Crl:CD1 (born and nurtured in our breeding facility) were housed in Makrolon cages under standard laboratory conditions (room temperature 21 ± 1°C [mean ± SEM]; relative humidity 40–55%; photoperiod 12L:12D), supplied with a standard breeding diet (V1126, Ssniff GmbH, Germany) and tap water *ad libitum*. Cages were equipped with bedding material (Lignocel, J. Rettenmaier & Söhne GmbH, Germany, heat treated) and enriched with cardboard tubes (SDS Deutschland c/o Jung GmbH, Germany) and nesting material (Pur-Zellin; Paul Hartmann AG, Germany). SPF quality of the animals was confirmed by a sentinel program according to FELASA recommendations [25]. The study was discussed and approved by the ethics and animal welfare committee of the University of Veterinary Medicine Vienna in accordance with Good Scientific Practice (GSP) guidelines and national legislation.

### Preliminary study

To test the potential effects of experimental housing conditions during fecal sample collection we conducted a preliminary experiment with four eight-week-old B6 and CD1 female mice. Before fecal collection started animals of each strain were grouped together for a one week long adaptation period in our experimental facility. They were then housed individually for the sampling period of seven days. During this time the mice were kept on cotton sheets without wood bedding. In order to identify the most optimal day for unbiased FCM measurement following separation, we collected feces once daily beginning on the next day post separation (9 a.m.) and continued for seven consecutive days, then samples were stored frozen (-20°C) until analysis.

### Main study

Two groups of ten B6 and ten CD1 female mice were delivered from our breeding facility to the experimental facility at the age of three weeks. After a one week adaptation period mice were separated for the first time at 11 a.m. into type II cages lined with cotton sheets. Based on results of our preliminary study (see results of the preliminary study), voided feces collection started on day five after separation of animals, at the following sampling intervals: 3 p.m., 7 p.m., 11 p.m., 3 a.m., 7 a.m. and 11 a.m. (every 4 hours), then stored frozen at -20°C. Afterwards, the mice were returned to their home cages and were housed in groups until the next sampling date. Sampling was performed at the age of 1 month, 2 months and then repeated at every second month until the last of the surviving mice reached the age of 26 months (one CD1 mouse and five B6 mice). This sampling interval yielded a maximum of 84 samples per mouse.

Clean fecal samples of each mouse from each sampling interval were dried, homogenized and 0.05 g of the dry weight (DW) was extracted with 1 ml of 80% methanol. Concentrations of FCM were analyzed by an in-house 5α-pregnane-3β,11β,21-triol-20-one enzyme immunoassay (EIA) that was developed and successfully validated for measuring corticosterone metabolites in mice. For further details of the EIA see [3, 6].

### Statistical analysis

For our preliminary study we used ANOVAs with repeated measures, followed by simple linear contrasts with Bonferroni’s alpha correction as a post hoc procedure, to estimate the effects of separation on FCM concentrationsover the seven day pre-study period following separation.

For the main study we analyzed the effects of age and seasons on mean FCM concentrations, as well as the coefficient of variation (CV) by using linear mixed effects models for each strain. The daily variation of FCM was estimated by calculating the CV. The CV was determined as ratio of the standard deviation to the mean of measured FCM concentration for each time point. Due to the loss of data points over the 26 months period, we assumed for each model a heterogeneous first order autoregressive (ARH1) covariance matrix as error structure, where the individuals (id) were included as a random factor. In addition, a linear regression analysis was performed for age in order to describe the development of FCM concentration over the life-time of mice. Multiple comparisons were only conducted between seasons (sampling points) using Bonferroni’s alpha correction procedure. Shapiro-Wilk-test was performed to test the assumption of normal distribution of mean FCM and CV.

All statistical analyses were performed using IBM SPSS v19. Tests are two-sided and a p-value of ≤ 5% (*, p≤0.05) was considered significant.

## Results

### Preliminary study

**Fig 1:**
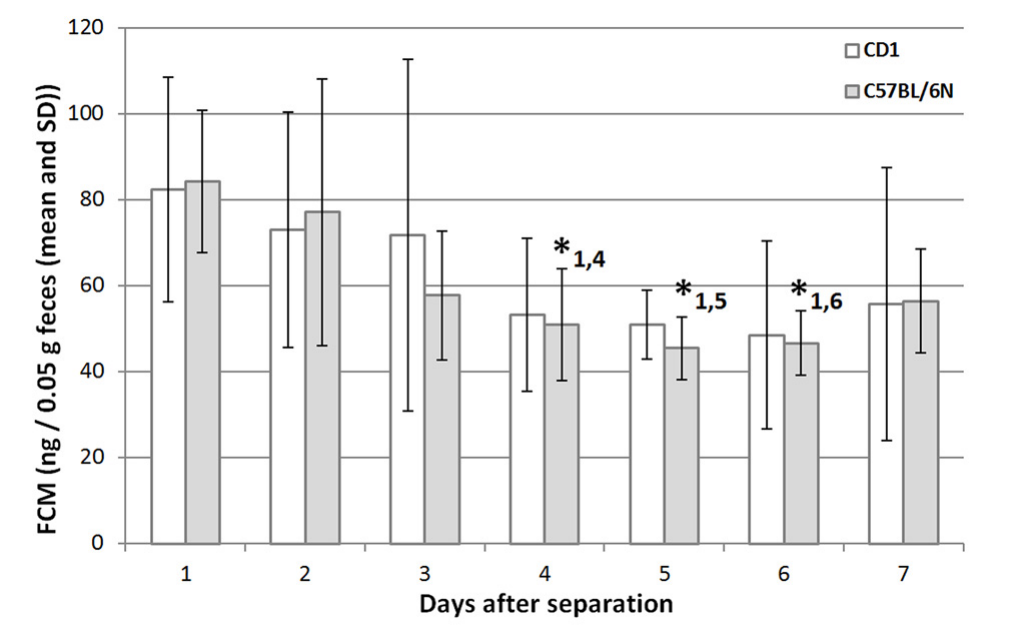
Concentrations of fecal corticosterone metabolites (FCM; mean ± SD) of female C57BL/6N and Crl:CD1 mice over a seven day period after separation. Preliminary study (n = 4; each strain). *, p≤0.05 for paired samples t-tests between day 1 and days 4, 5 and 6.

Measured concentration of FCM decreased from day 1 post separation to day 3, suggesting an adaptation to the individual housing conditions. From day 4–7 these measurements leveled off at lower concentrations in both strains (Fig 1). No significant differences were measured on a daily basis between samples of CD1 mice obtained during the seven-day test period. In contrast, B6 mice showed a significant decrease of FCM concentration between day 1 after separation compared to days 4, 5 and 6, however, no significant differences were found between the FCM concentrations of day 3 and the following days. We arbitrarily selected day 5 after separation as most appropriate for the commencement of our 24h-sampling period to obtain FCM concentrations for the main study.

### Main study

**Fig 2:**
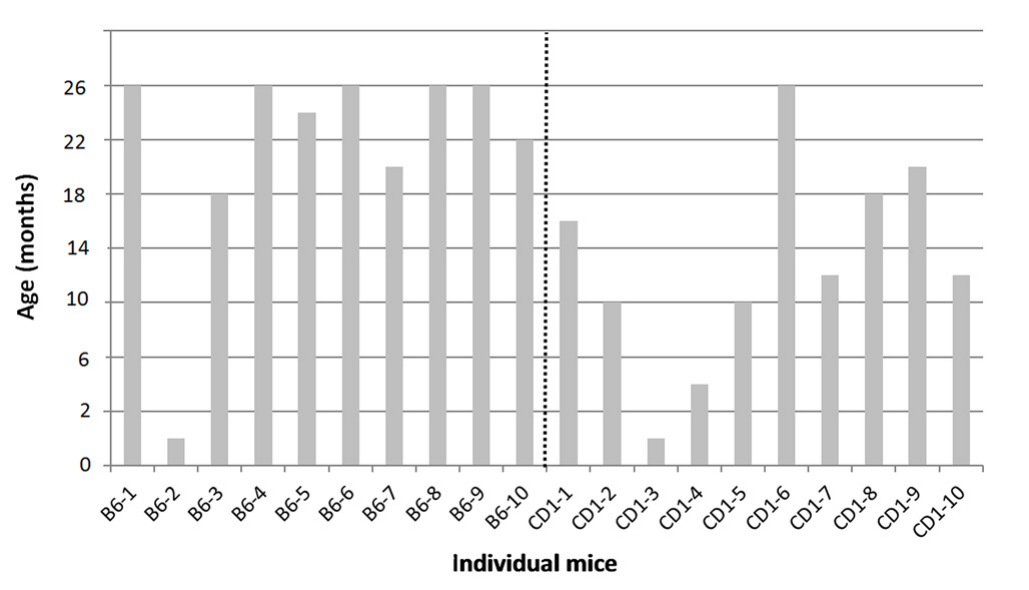
Overview of maximum ages and sampling points for individual female mice of the C57BL/6N and Crl:CD1 strain mice in main study.

To address any possible impact of genetic differences we analyzed two commonly used mouse strains. Sampling of feces was performed based on the described experimental protocol until the maximum age of 26 months. Several mice from each group have died spontaneously in the course of our study. Surprisingly, the survival rate of animals of the B6 inbred strain was generally higher compared to the CD1 outbred mice (Fig 2). All but one B6 mice survived until the 10^th^ collection time point, therefore, most of the test animals of this strain reached the minimum of 18 months in age. In contrast, only six of 10 CD1 outbred mice reached the age of 12 months, only three survived to the age of 18 months and only one animal lived to the maximum of 26 months. The short lifetime of CD1 mice resulted in a substantial reduction of sample size for the CD1 group that limited the statistical outcome for the period after 12 months of age.

**Fig 3:**
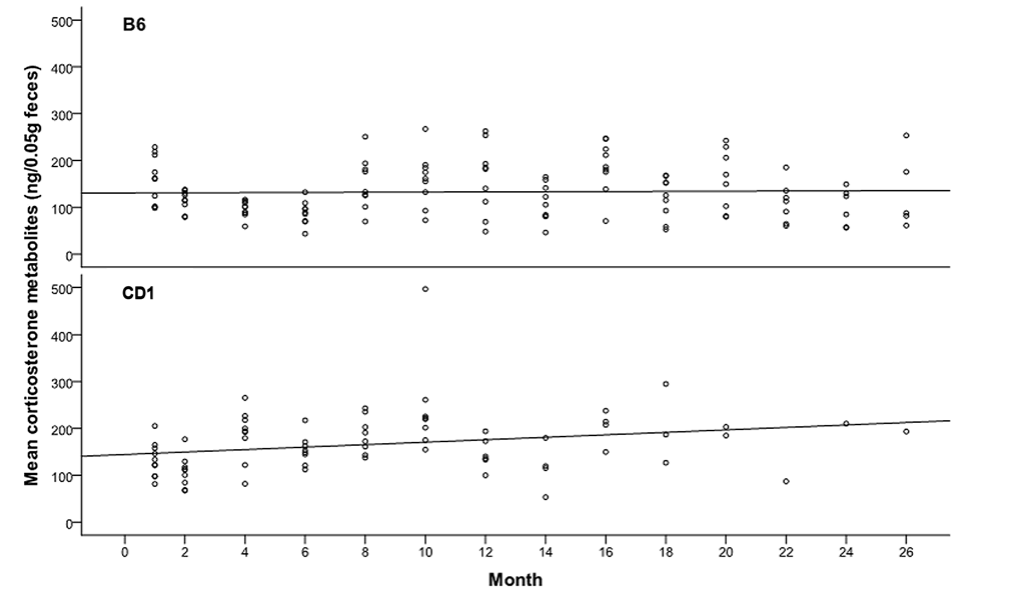
Mean concentrations of fecal corticosterone metabolites (FCM) per mouse and corresponding sampling points. A linear regression analysis describes the slope of FCM concentration over lifetime.

**Fig 4:**
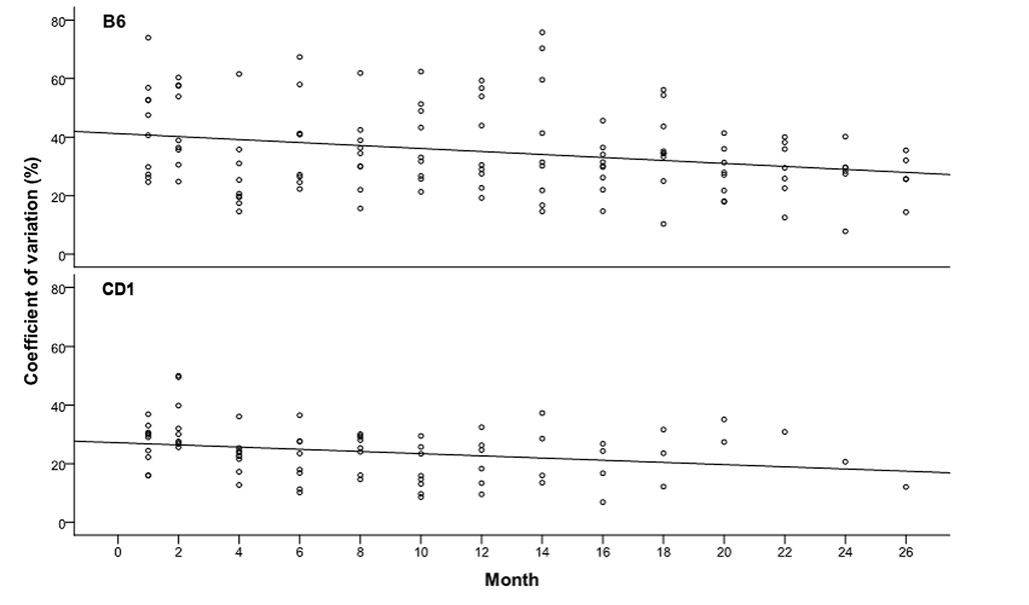
Diurnal rhythmicity calculated separately per mouse and sampling point as coefficient of variation (CV). A linear regression analysis describes the slope of circadian oscillation of FCM concentrations over lifetime.

Figs 3 and 4 provide an overview of the data measured over all time points presenting mean FCM concentrations and CV. Daily mean values of individual FCM concentrations of CD1 mice increased over the test period from 80–200 to 130–500 ng/0.05 g feces (Fig 3). In contrast, the mean values in B6 mice stayed nearly constant over their lifetime and varied in the range of 60–250 ng/0.05 g feces (Fig 3). The results of the mixed effects model analyses are presented in Table 1.

**Table 1:** Main results of the mixed effects model analysis showing impact of age and seasons on mean FCM concentration and CV. Slopes of FCM concentration and CV over progression of age are given as a result of a linear regression model.

	Mixed model using animal id as a random factor								Linear regression	
Strain	Dependent	Factor	Var(id)%^a^	AIC^b^	F	Df numerator	Df error	p	slope	p
**B6**	**Mean**	**Age**	32	1069,8	7,2	13	14,2	0.001	0.29	0.829
		**Season**	23.3	1157.6	5.18	6	26	0.001	-	-
	**CV**	**Age**	6.0	90.9	2.9	13	12.4	0.035	-0.010	0.004
		**Season**	23.8	123.8	2.7	6	7.4	0.022	-	-
**CD1**	**Mean**	**Age**	4.0	648.2	6.04	11	9.6	0.005	6.97	0.001
		**Season**	21.3	375.1	9.7	6	29	0.001	-	-
	**CV**	**Age**	12.5	101	3.3	11	7.8	0.055	-0.008	0.030
		**Season**	12.5	112.5	4.6	6	14	0.009	-	-

^a^Variance component in percent caused by the animal id.

^b^Akaike’s information criterion.

Diurnal variation calculated as CV was more distinct in the B6 strain compared to CD1 mice (Fig 4). Although, we observed a strong variation of FCM concentrations between animals on each sampling date, the corticosterone metabolite excretion followed the typical pattern of diurnal periodicity in almost all animals (data not shown). This circadian oscillation, however, decreased continuously with the advancement of age in both strains (Fig 4).

**Fig 5:**
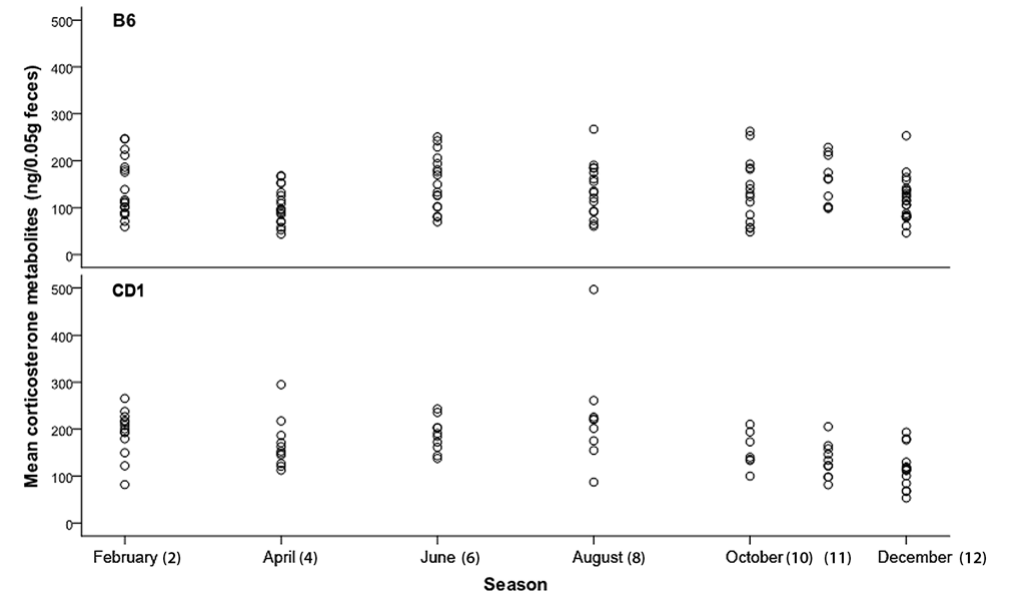
Mean concentrations of fecal corticosterone metabolites (FCM) per mouse and lifetime, pooled for the corresponding seasonal sampling points.

To evaluate any possible seasonal effects on FCM concentration, data of the same month (time point) of a year were pooled from the complete 26 month test period for each strain. Circannual changes were found for both strains. Differences were observed more often between seasons (sampling points) of short days with sparse daylight versus seasons with longer daylight hours. Significant differences in pairwise comparisons of FCM concentration at different time points were calculated for B6 mice: 2/6 p = 0.034; 4/6 p = 0.026; and for CD1 mice: 2/11 p = 0.025; 2/12 p = 0.001; 6/11 p = 0.025; 6/12 p = 0.001 (Fig 5).

## Discussion

We demonstrate here for the first time an age related effect on the concentration of corticosterone metabolites in feces of laboratory mice. Starting with comparable concentrations as juveniles the level of FCM stayed constant in B6 animals over a 26-month period, but continuously increased in CD1 mice over their lifetime. This age dependent increase resulted in a strain specific difference of the FCM concentration. Furthermore, the typical circadian oscillation of corticosterone metabolite concentrations decreased in both strains over the test period.

Stress hormone metabolites in the feces proved to be an important tool for non-invasive assessment of distress in animals of different species [26]. In order to estimate the level of experimental burden in laboratory mice we and others have utilized assaying of FCM concentration as a refined alternative to invasive blood serum sampling and analysis [27–32]. If the data collection is restricted to defined experimental time points or short periods, results will not be severely impacted by age related effects. However, in case of long-term studies the question arises if changes in hormone metabolite levels are also affected by aging. Furthermore, data comparison between studies with animals of different ages might be prone to an age related bias.

We addressed this problem by measuring FCM levels of inbred and outbred female mice without any experimental treatment over a period covering almost the complete lifetime of a laboratory mouse. For mice it is well known that patterns of FCM excretion differ significantly between sexes [3]. Nevertheless, for animal welfare reasons we conducted the study exclusively on females because repeated separation and regrouping of male mice is not advisable. The necessary individual housing of males over a long period of time would result in chronic distress due to social isolation, which may result in false measurements of stress hormones that reflect the effects of isolated housing [33].

The continuous increase of FCM concentrations in CD1 mice compared to the steady values measured for B6 mice demonstrated strain specific variability (Fig 3). Species differences in glucocorticoid secretion and metabolite excretion are well described [2, 26]. Our results indicate that even different strains of the same species might show differences in their adrenocortical activity. Reports about strain specific differences regarding stress hormone secretion are rare and inconsistent. Jones and co-workers found differences between C57BL/6 and DBA/2 mice regarding basal levels of plasma corticosterone and in response to stressors [8]. In contrast, the analysis of FCM of untreated C57BL/6J and C3H/HeJ mice revealed similar concentrations for both strains but strain specific differences in response to surgical stress and pain [34]. It should however be noted that both studies considered stress hormone values at a specific age of the animals, which may indeed be different at any given point in time depending on a mice’s age, as it has been demonstrated by our results.

Basal levels of plasma corticosterone showed no differences between young and aged F344/N and Long-Evans rats [35, 36]. However, in response to acute stress, levels increased and took significantly longer to return to baseline in aged rats. This prolonged stress response could be explained by the observed age-related down regulation of glucocorticoid receptors in several brain structures [35, 36]. The FCM assay used in the presented study is not a snap-reading method but rather integrate hormone secretion over a period. A protracted elevation of corticosterone after stress, due to failure of the negative feedback response of the HPA axis, could thereby result in an elevated FCM concentration. The frequent occurrence of spontaneous cases of death in the CD1 strain significantly reduced sample size of the present study, especially for the age groups older than 12 months. However, this unexpected outcome may reflect a bad general constitution among animals of this outbred stock, resulting in a stressful period (measured as continuously increasing FCM concentration) towards the end of their life.

Although care was taken to standardize the procedure of feces collection, a symptomatic high level of variation in FCM levels was seen within both mouse strains of the study. Nevertheless, the diurnal changes of FCM followed the typical excretion profile as described before [6]. The results of our study clearly demonstrate a decreasing oscillation of FCM concentration towards the end of life for both analyzed strains (Fig 4). This is consistent with the previously reported modest circadian variation of plasma corticosterone levels for animals of the oldest group of 3-, 9- and 16-month-old male C57BL/6J mice [37]. Interestingly, our observations also follow the results of a study on diurnal cortisol profiles in human adults in which the likelihood for a flattened profile (in relation to the normative profile) was increased in older subjects with reportedly poorer health conditions [38, 39].

Disturbance of the cortisol rhythm is a common symptom for patients suffering from major depression [40, 41]. In addition to its impact on several regions of the central nervous system, glucocorticoids also play a key role in regulating appropriate circadian clock mechanisms in peripheral tissues and organs [42]. In CD1 mice it has been shown that the daily rhythm of glucocorticoids is strongly involved in synchronization of the liver circadian transcriptome [43]. Since changes in biological rhythms could be a sensitive indicator for impaired health and wellbeing, the level of stress hormone secretion, supplemented by its circadian patterning, can improve assessment of an individual’s condition [44].

The here presented long-term study also enabled us to investigate any seasonal effects on FCM concentration. Seasonal rhythmicity for glucocorticoid concentrations have been reported for free-living species of different taxa [45]. This is not surprising because factors like changing climate conditions, periods of competition for food or mating partners and the reproductive status of females are likely to influence release of the stress hormones. The circadian regulation of corticosteroid secretion does not depend on the rhythmic release of ACTH but rather results from diurnal variation in adrenal responsiveness to ACTH [46, 47]. This intrinsic rhythm of response is activated by light stimuli via the suprachiasmatic nucleus and the sympathetic nervous system. Interestingly, up to an intensity of 40 lux, light increased the corticosterone level in a dose dependent manner [46].

By comparing the measured FCM data between predetermined time points during the seasons we were able to identify specific variations. Significant differences were more distinct between seasons of short days with sparse daylight (winter) and seasons with longer daylight hours (summer) in both strains (Fig 5). Considering the fact that such important seasonal factors as changes in light-dark cycles, light intensity, room climate, food supply and food quality, or variation in the breeding seasons can be excluded in our study, we can only speculate that (domesticated) laboratory mice may still be subjected to intrinsic mechanisms. These mechanisms have evolutionary significance in that they enable an organism to alleviate repeatedly stressful situations in its natural habitat. In other words, circannual rhythm of corticosteroid secretion could be internalized like circadian oscillation which also stays active even if external conditions are constant (for example constant darkness). Therefore, the possibility of seasonal effects on stress hormone secretion should be considered in spite of highly standardized environmental conditions of laboratory animal facilities.

In summary, measurement of fecal corticosterone metabolites over the lifetime of untreated mice revealed a significant impact of age on hormone levels and on its circadian changes. The age-related impact was strain specific for mean FCM values over the test period but identical for both strains in form of a continuous flattening of the diurnal rhythm with increasing age. Therefore, mouse strain, age of animals and circannual rhythmicity all should be taken into consideration as possible modifying factors to improve comparability and reproducibility of FCM data measured in animal experiments.
